# Expression of *Fused in sarcoma* mutations in mice recapitulates the neuropathology of FUS proteinopathies and provides insight into disease pathogenesis

**DOI:** 10.1186/1750-1326-7-53

**Published:** 2012-10-10

**Authors:** Christophe Verbeeck, Qiudong Deng, Mariely DeJesus-Hernandez, Georgia Taylor, Carolina Ceballos-Diaz, Jannet Kocerha, Todd Golde, Pritam Das, Rosa Rademakers, Dennis W Dickson, Thomas Kukar

**Affiliations:** 1 Department of Neuroscience, Mayo Clinic, Jacksonville, FL, USA; 2 Department of Pharmacology and Neurology, Emory University School of Medicine, Atlanta, GA, USA; 3 Center for Translational Research in Neurodegenerative Disease, College of Medicine, University of Florida, Gainesville, FL, USA; 4 Department of Human Genetics, Emory University School of Medicine, Atlanta, GA, USA

**Keywords:** Amyotrophic lateral sclerosis, Frontotemporal lobar degeneration, Fused in sarcoma proteinopathies, Transgenic mouse models, Adeno-associated virus, Neuronal cytoplasmic inclusions, Ubiquitin, p62/SQSTM1, α-internexin, PABP-1, Stress granules, RNA dysfunction

## Abstract

**Background:**

Mutations in the gene encoding the RNA-binding protein fused in sarcoma (FUS) can cause familial and sporadic amyotrophic lateral sclerosis (ALS) and rarely frontotemproal dementia (FTD). FUS accumulates in neuronal cytoplasmic inclusions (NCIs) in ALS patients with FUS mutations. FUS is also a major pathologic marker for a group of less common forms of frontotemporal lobar degeneration (FTLD), which includes atypical FTLD with ubiquitinated inclusions (aFTLD-U), neuronal intermediate filament inclusion disease (NIFID) and basophilic inclusion body disease (BIBD). These diseases are now called FUS proteinopathies, because they share this disease marker. It is unknown how FUS mutations cause disease and the role of FUS in FTD-FUS cases, which do not have FUS mutations. In this paper we report the development of somatic brain transgenic (SBT) mice using recombinant adeno-associated virus (rAAV) to investigate how FUS mutations lead to neurodegeneration.

**Results:**

We compared SBT mice expressing wild-type human FUS (FUS_WT_), and two ALS-linked mutations: FUS_R521C_ and FUS_Δ14_, which lacks the nuclear localization signal. Both FUS mutants accumulated in the cytoplasm relative to FUS_WT_. The degree of this shift correlated with the severity of the FUS mutation as reflected by disease onset in humans. Mice expressing the most aggressive mutation, FUS_Δ14_, recapitulated many aspects of FUS proteinopathies, including insoluble FUS, basophilic and eosiniphilic NCIs, and other pathologic markers, including ubiquitin, p62/SQSTM1, α-internexin, and the poly-adenylate(A)-binding protein 1 (PABP-1). However, TDP-43 did not localize to inclusions.

**Conclusions:**

Our data supports the hypothesis that ALS or FTD-linked FUS mutations cause neurodegeneration by increasing cyotplasmic FUS. Accumulation of FUS in the cytoplasm may retain RNA targets and recruit additional RNA-binding proteins, such as PABP-1, into stress-granule like aggregates that coalesce into permanent inclusions that could negatively affect RNA metabolism. Identification of mutations in other genes that cause ALS/FTD, such as C9ORF72, sentaxin, and angiogenin, lends support to the idea that defective RNA metabolism is a critical pathogenic pathway. The SBT FUS mice described here will provide a valuable platform for dissecting the pathogenic mechanism of FUS mutations, define the relationship between FTD and ALS-FUS, and help identify therapeutic targets that are desperately needed for these devastating neurodegenerative disorders.

## Background

Mutations in the *Fused in Sarcoma* (*FUS*) gene were recently discovered in some cases of familial and sporadic amyotrophic lateral sclerosis (ALS) and more rarely fronto-temproal dementia (FTD) [1-3]. FUS is a 526 amino acid DNA/RNA binding protein member of the FET family (FUS/Ewing’s sarcoma/TATA-binding protein-associated factor) [4]. The FUS gene, also known as TLS (*translated in sarcoma*), was first described as a N-terminal fusion that produced hybrid oncogenes [5]. The full length FUS protein is now appreciated to play a role in a number of critical cellular functions, including gene expression, RNA processing, RNA transport, and genomic integrity [5,6]. The highest levels of FUS are found in the nucleus, driven by a highly conserved carboxyl (C) terminal PY nuclear localization signal (PY-NLS) [7]. FUS is found in the cytoplasm at lower levels and can shuttle rapidly between the nucleus and the cytoplasm [8,9]. The majority of disease-linked FUS mutations cluster in the C-terminus and disrupt nuclear import, but the precise pathogenic mechanism of FUS mutations is currently unknown.

The identification of FUS mutations and accumulation of FUS within ubiquitin-positive neuronal cytoplasmic inclusions (NCI) in a portion of ALS cases led to the re-examination of other neurological diseases with NCI of unknown origin. Subsequently, abnormal FUS was detected within NCI, as well as glial inclusions, in several uncommon forms of frontotemporal lobar degeneration (FTLD), which is the term for the pathology underlying the clinical syndrome FTD. These rare subsets of FTLD were previously referred to as atypical FTLD with ubiquitinated inclusions (aFTLD-U), neuronal intermediate filament inclusion disease (NIFID) and basophilic inclusion body disease (BIBD) [10]. These disorders have now been grouped together as the FUS proteinopathies, because they share a common pathology and a presumed underlying disease mechanism [10]. The two major clinical and pathological types are known as frontotemporal lobar degeneration with FUS pathology (FTLD-FUS) and ALS with FUS pathology (ALS-FUS). This nomenclature is analogous to the classification that has been developed for the TDP-43 proteinopathies (ALS-TDP and FTLD-TDP), which have inclusions that contain the RNA-binding protein TDP-43 protein [11].

In this report we have utilized a technique called somatic brain transgenesis (SBT) to investigate how FUS mutations lead to neurodegeneration. SBT uses recombinant adeno-associated virus (rAAV) to express a cDNA predominantly in neurons throughout much of the brain for the lifetime of the mouse, beginning a few weeks after birth [12,13]. We compared over expression of wild-type human FUS (FUS_WT)_, and two mutations associated with ALS: FUS_R521C_, or FUS_Δ14_. Expression of both FUS mutants led to increased FUS protein in the neuronal cytoplasm, the degree of which correlated with the severity of the mutation as reflected by disease onset in humans. Mice expressing the most aggressive mutation, FUS_Δ14_, recapitulated many aspects of human FUS proteinopathies, including insoluble FUS protein, basophilic and eosiniphilic neuronal cytoplasmic inclusions (NCI), and presence of other pathologic markers, including ubiquitin, p62/SQSTM1, α-internexin, and the polyadenylate-binding protein 1 (PABP-1).

## Results

### Generation of mice overexpressing FUS using somatic brain transgenesis (SBT)

**Figure 1:**
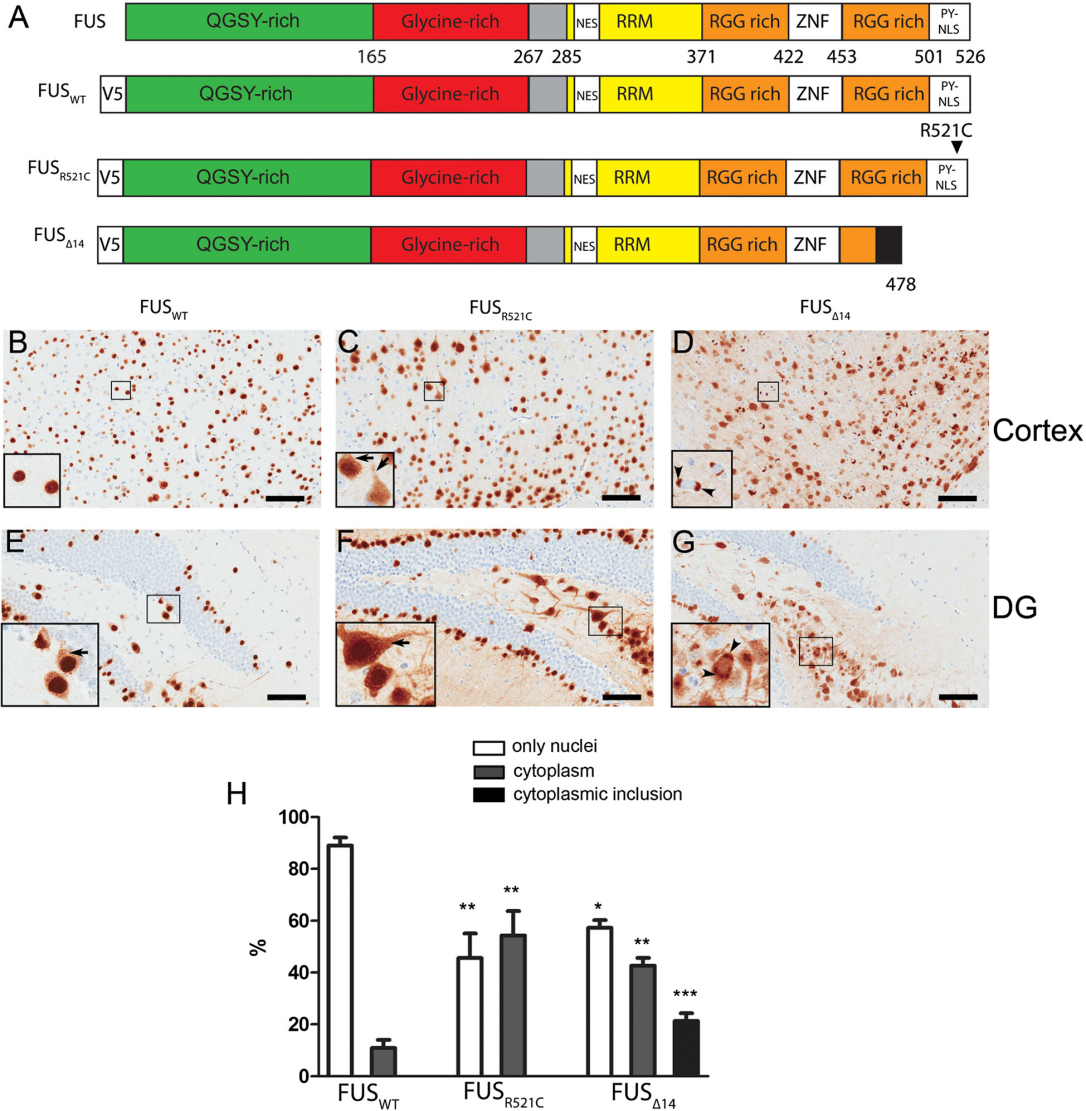
**Generation of Human *Fused in Sarcoma *(FUS) mouse models using rAAV1 and SBT.** (**A**). Diagram of FUS_WT_, FUS_R521C_, and FUS_Δ14_ expression constructs. All FUS constructs were cloned with a V5 epitope tag on the amino-terminus to aid immunodetection. The major protein domains of FUS are highlighted. QGSY=Gln-Gly-Ser-Tyr rich region. Glycine rich region. NES=nuclear export signal. RRM= RNA recognition motif. RGG= Arg-Gly-Gly-rich motif. ZNF=zinc finger motif. PY-NLS=Pro-Tyr nuclear localisation signal. (**B**) to (**G**). Immunohistochemistry (anti-V5 antibody) detects widespread expression of FUS_WT_, FUS_R521C_, and FUS_Δ14_ in cerebral cortex and dentate gyrus (DG). Wild type FUS is mainly localized in the nuclei (**B** insert). Intense nuclear V5 staining in cerebral cortex (**B**) and weak cytoplasmic v5 staining in DG (**E**). In the FUS_R521C_ model, FUS is no longer predominantly located in the nucleus, but also found in the soma and dendrites (**C** and **F**). FUS_Δ14_ mice have a dramatic translocation of FUS from the nucleus to the cytoplasm, and formation of neuronal cytoplasmic inclusions (NCIs) (**D** and **G**). *Scale bar:* 100 μm (**H**). Histogram showing the percent of nuclear and cytoplasmic v5 staining,cytoplasmic inclusion in cerebral cortex. (n=4; S.E.M.) * P<0.05, **P<0.01 and ***P<0.001, one way ANOVA.

We utilized SBT to express wild type human FUS and two FUS mutations associated with ALS in the brains of mice to investigate the role of FUS in neurodegeneration using an *in vivo* model. In this experimental paradigm, newly born (P0) litters of mice were administered recombinant AAV1 encoding FUS_WT_, FUS_R521C_, or FUS_Δ14_, through bilateral intracerebroventricular injection. The FUS R521C mutation, which has been identified in 16 ALS families to date, occurs within the PY nuclear localization signal (PY-NLS) region, and results in an average age of onset of 40 years [1,14,15]. The third model, FUS_Δ14_,was based on a *de novo* mutation found in a patient with sporadic ALS that we reported previously [16]. Briefly, a mutation in intron 13 of the *FUS* gene (g.10747A>G) causes skipping of exon 14, a frame shift, and premature termination in exon 15, leading to a truncated FUS protein of 478 amino acids that lacks the C-terminal PY-NLS (Figure 1A). This mutation (FUS_Δ14_) is associated with early disease onset (20 years) and a rapid disease progression (22 months).

### Characterization of pathology in FUS mice

**Figure 2:**
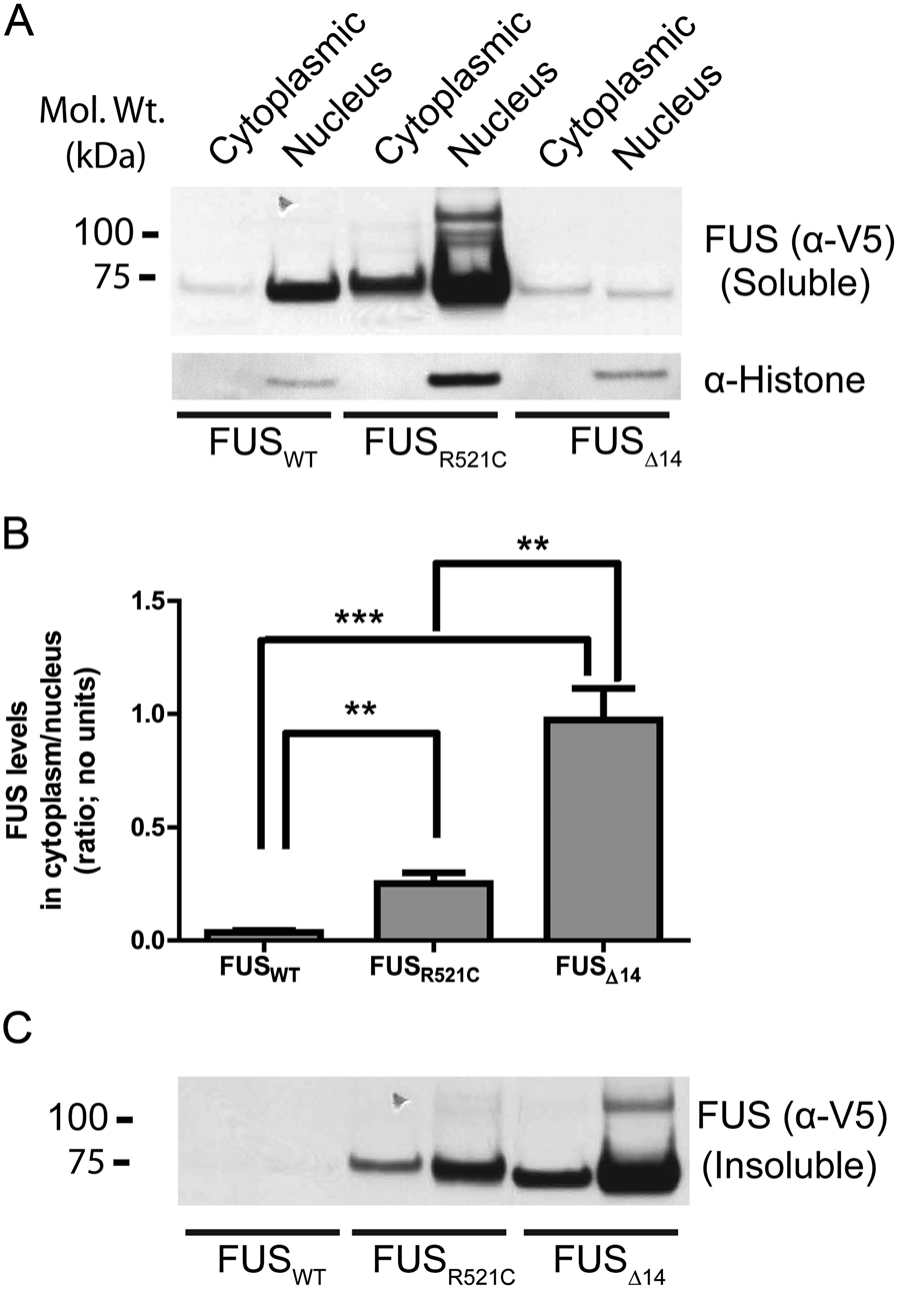
**FUS mutations cause an aberrant subcellular redistribution in mouse neurons.** A representative immunoblot (**A**) of the V5 tagged FUS proteins extracted from AAV injected mouse brains. Tissue extracts from FUS_WT_, FUS_R521C_, and FUS_Δ14_ brain were separated into soluble fractions from the cytoplasm and nucleus. Histone 3 staining was used as a nuclear marker to verify extraction fidelity. (**B**) The ratio of cytoplasmic FUS to nuclear FUS was calculated based on quantification of immunoblots for different FUS constructs (n=4; S.E.M.). A higher ratio of FUS_R521C_ and FUS_Δ14_ are found in the cytoplasm. ** P<0.01 and ***P<0.001. (**C**) FUS_R521C_ and FUS_Δ14_ protein are more insoluble than FUS_WT_.

Three months after viral injection, mice were killed and one brain hemisphere was fixed for neuropathologic characterization, while the other hemisphere was flash frozen for biochemical fractionation. Mice appeared healthy at the time of death and did not display obvious motor impairment or an abnormal grasping phenotype (data not shown). FUS constructs contained a V5 epitope tag on the amino-terminus to aid visualization of protein expression and do not interfere with protein function or cellular localization [16]. Based on V5 immunohistochemistry FUS_WT_, FUS_R521C_, and FUS_Δ14_ mice had widespread FUS protein expression, throughout the brain, with the highest levels in the cerebral cortex and the hippocampus (Figure 1B-G). Using the SBT paradigm transgene expression is neuronal with no detectable glial expression, as assessed by double immunofluorescence (Additional file 1: Figure S1). Neurons expressing FUS_WT_ showed predominantly nuclear localization, with low, but detectable, levels of cytoplasmic protein based on immunohistochemistry and subcellular fractionation (Figure 1 and 2). FUS_R521C_ mice had marked increases in FUS immunoreactivity in the neuronal cytoplasm. The presence of nuclear FUS_R521C_ was a consistent feature; however, FUS_R521C_ was also detected in the soma, dendrites, and axons of neurons in mice, especially in the hippocampus (Figure 1C and F). Despite increased cytoplasmic levels of FUS_R521C_, no obvious inclusions or aggregates of FUS were observed in mice injected with FUS_WT_ or FUS_R521C_. FUS_Δ14_ mice showed the greatest cytoplasmic redistribution, with some neurons showing no nuclear FUS reactivity but strong labelling of the cell body and processes in cortex. A portion of neurons in FUS_Δ14_ mice contained FUS-positive neuronal cytoplasmic inclusions (NCIs), which bared striking resemblance to the NCIs that are a characteristic pathologic feature of ALS and FTD-FUS (Figure 1D and 1G). In cortex, the percentage of transduced neurons with cytoplasmic distribution of FUS significantly increased in FUS_R521C_ and FUS_Δ14_ mice (Figure 1H). The FUS_Δ14_ mice were the only group that had NCI, reaching ~20% of neurons expressing FUS (Figure 1H). Mutation-dependent FUS redistribution also was confirmed by double labelling with V5 and a neuronal marker (Additional file 2: Figure S2). Despite the presence of NCI, we did not observe any obvious neuronal loss or degeneration when examining haematoxylin and eosin (H&E) stained sections. Activated caspase-3 and TUNEL assays were also negative (data not shown), suggesting that apoptosis is not occurring in FUS mice at this age. Further, we did not observe marked astrocytosis or microglial activation at this age (Additional file 3: Figure S3).

We next examined the biochemical solubility of the different FUS proteins expressed in the SBT mice. The cytoplasm and intact nuclei were isolated from the frozen brain hemisphere of the FUS mice analysed above using immunohistochemistry (Figure 1). Nuclei were lysed in detergent to isolate the soluble nuclear fraction. The cytoplasmic and nuclear extracts were centrifuged at 20,000 x g to pellet insoluble proteins. Samples were separated by SDS/PAGE, analysed on immunoblots, and quantified using densitometry (Figure 2A). Comparison of the ratio of soluble cytoplasmic to nuclear FUS protein using densitometry confirmed that the steady state levels of the FUS mutants are higher in the cytoplasm, with FUS_Δ14_ showing the strongest shift (Figure. 2A and B). Intriguingly, the levels of FUS_Δ14_ detected in the cytoplasmic and nuclear lysates did not appear to match the immunohistochemistry (Figure 1B-H) that suggested robust expression of all FUS constructs. We then analysed the pellets resulting from the protocol used biochemical isolation of the cytoplasm and nucleus, and discovered that the majority of FUS_Δ14_ protein partitions into the insoluble fraction (Figure 2C). No FUS_WT_, but a portion of FUS_R521C_ protein was also detected in the insoluble fraction.

### Formation of neuronal cytoplasmic inclusions in FUS_Δ14_ mice

**Figure 3:**
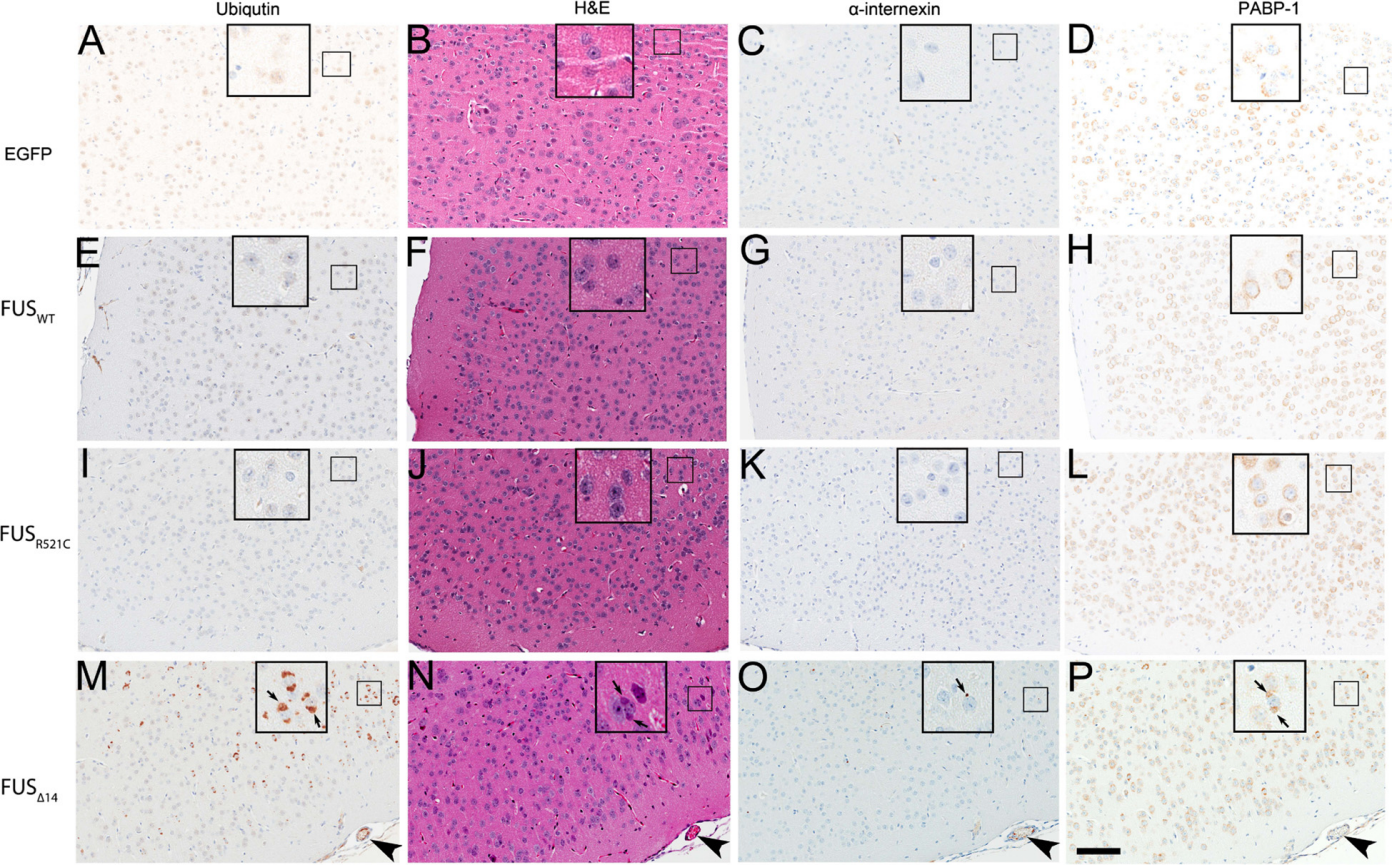
**Neuropathology of SBT FUS**_**Δ14**_**mice is similar to human FUS proteinopathies (A-P).** Adjacent sections from the cerebral cortex of eGFP (**A**-**D**), FUS_WT_ (**E**-**H**), FUS_R521C_ (**I**-**L**), and FUS_Δ14_ (**M**-**P**) mice stained with protein NCI markers found in aFTLD-U, NIFID, BIBD and hematoxylin-eosin (**H**&**E**). (**M**) Only FUS_Δ14_ brains contain ubiquitinated NCIs. (**N**) Hematoxylin-eosin (**H**&**E**) staining of FUS_Δ14_ cerebral cortex shows cytoplasmic basophilic inclusions (arrows). (**O**) NCIs are infrequently positive for α-internexin, similar to the pathological NCIs found in NIFID cases. (**P**) Some NCI in the cerebral cortex of FUS_Δ14_ mice contain the stress granule marker protein PABP-1. *Scale bar*: 100 μm. Slides are orientated to a common blood vessel (arrow head) to serve as a landmark (**M**-**P**).

**Figure 4:**
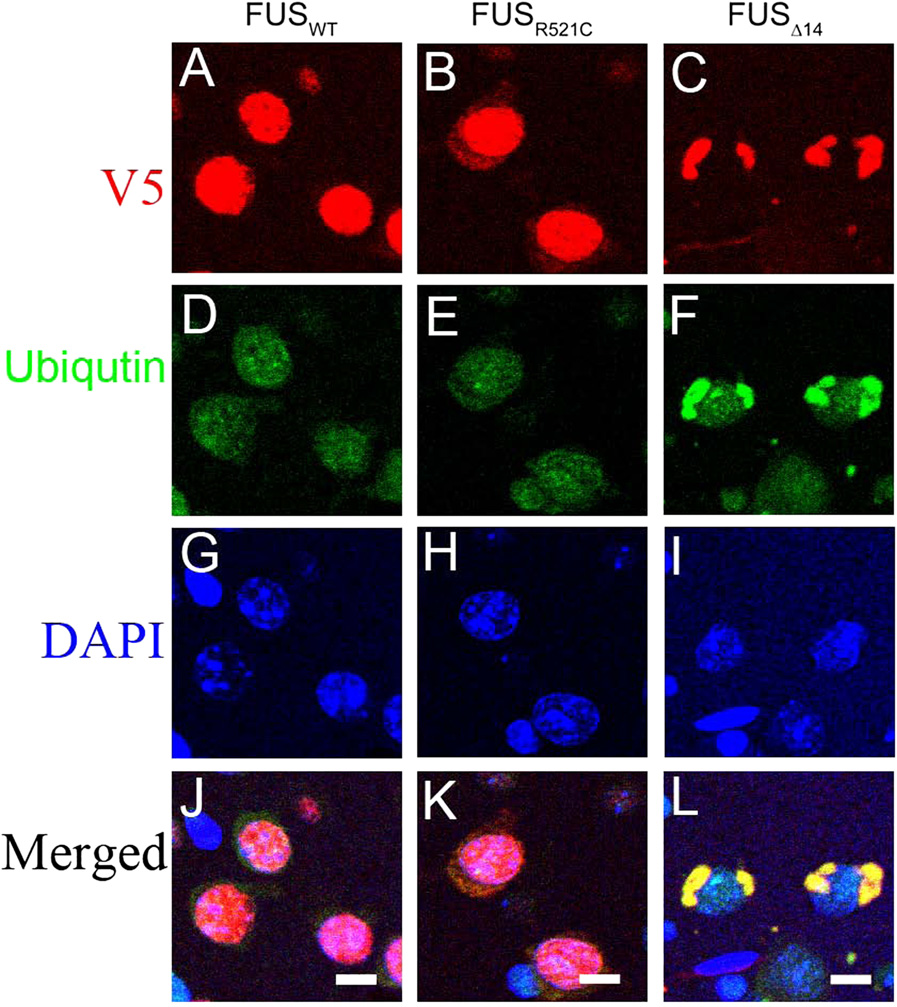
**Ubiquitin co-localizes to FUS-positive inclusions in hFUS**_**Δ14**_**mice.** Confocal image showing FUS located in nucleus in FUS_WT_ mice (**A**), increased cytoplasmic distribution of FUS in FUS_R521C_ mice (**B**), and accumulation of FUS into NCIs in FUS_Δ14_ mice (**C**) that co-labels with ubiquitin (**F** and **L**). Nuclei were counterstained with DAPI (**G**, **H** and **I**). FUS detected with anti-V5 (**A**, **B**, **C**). *Scale bar*: 10 μm.

We next examined FUS mice for the presence of neuropathologic markers found in ALS or FTD. Sections from eGFP, FUS_WT_ and FUS_R521C_ mice had diffuse ubiquitin staining with no detectable inclusions (Figure 3A, E and I). In contrast, FUS_Δ14_ mice had frequent ubiquitin-positive NCIs (Figure 3M and Additional file 4: Figure S4). NCIs varied in size from small puncta to large, round inclusions (Additional file 5: Figure S5). Double-label immunofluorescence confirmed that FUS_Δ14_ and ubiquitin co-localize to the same inclusion (Figure 4). We did not observe an increase in high molecular weight smearing of FUS, an indicator of poly-ubiquitination, on immunoblots of brain tissue of FUS_Δ14_ compared to FUS_WT_ (data not shown). Furthermore, immunoblots directly for poly-ubiquitin did not detect a difference between FUS_WT_, FUS_R521C_ or FUS_Δ14_ mice, suggesting that FUS is not robustly ubiquitinated (data not shown).

**Figure 5:**
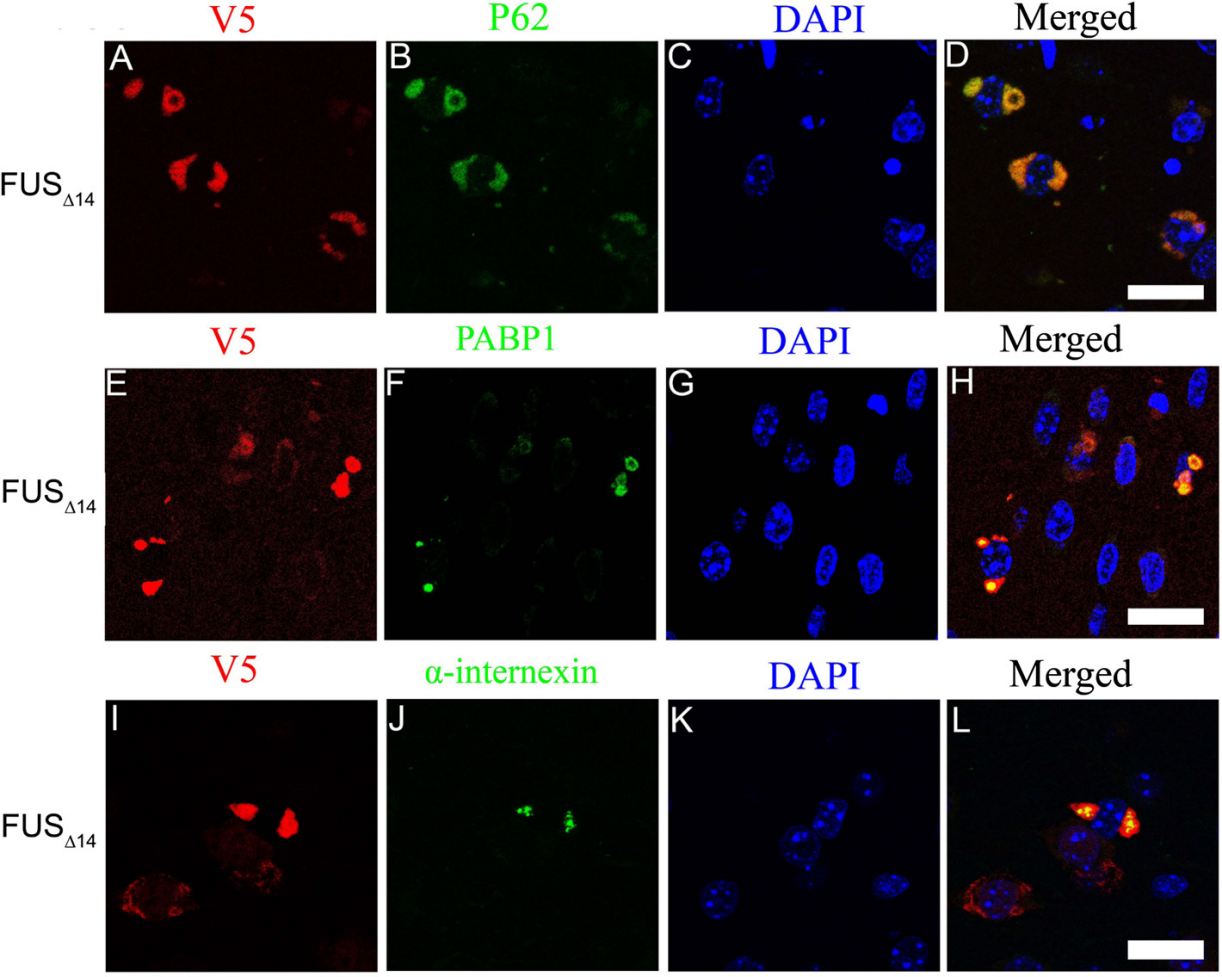
**Multiple neuropathologic markers co-accumulate in FUS**_**Δ14**_**mice NCIs.** Double labelling for FUS (anti-V5; **A**, **E** and **I**) and P62 (**B**), PABP1 (**F**) and α-internexin (**J**). Colocalization shown in merged image (**D**, **H** and **L**). Nuclei were counterstained with DAPI (**C**, **G**, and **K**). *Scale bar*: 20 μm.

Based on the robust formation of NCIs in the FUS_Δ14_ mice, we examined a panel of protein markers that were previously described in the NCIs of FUS proteinopathies. Many of the NCI in FUS_Δ14_ mice were basophilic by H&E staining, similar to the NCI found in BIBD, aFTLD-U, and NIFID cases (Figure 3N) [17-19]. Many of the basophilic inclusions were cytoplasmic, adjacent to the nucleus, and had a rounded, “Pick-body” like structure (Figure 3N insert). Eosinophilic staining was also noted on the periphery of inclusions, distinct spots within basophilic NCIs, or as distinct inclusions. We then stained for the neuronal intermediate filament protein α-internexin, which is one of the most abundant markers for NIFID [20]. A portion of the NCIs found in the FUS_Δ14_ model were positive for α-internexin (Figure 3O), but they were less frequent than NCIs labelled with ubiquitin (Figure 3M). NCI in FUS_Δ14_ were also immunoreactive for PABP-1, which was previously reported to stain NCI in BIBD and NIFID cases (Figure 3P) [21]. There was diffuse cytoplasmic staining of PABP-1 in eGFP, FUS_WT_ or FUS_R521C_ mice, but no obvious NCI (Figure 3D, H and L). Double-label immunofluorescence confirmed that α-internexin and PABP-1 co-localized with FUS inclusions in the FUS_Δ14_ model (Figure 5E-L).

We also examined the staining patterns of the p62/ sequestosome-1 (p62/SQSTM1) and optineurin (OPTN) proteins in FUS mice, which have been reported to be mutated in some familial and sporadic ALS cases [22,23]. P62 robustly co-localized with NCI in FUS_Δ14_ mice (Figure 5A-D). OPTN immunostaining was diffuse and widespread in the neuronal cytoplasm of FUS_WT_, FUS_R521C_, as well as FUS_Δ14_ mice. We found occasional increased OPTN staining in the neuronal cytoplasm in brain regions of FUS_Δ14_ mice with ubiquitin positive NCI, but no definite labelling of NCIs (Additional file 6: Figure S6).

### No redistribution or accumulation of TDP-43 in FUS mice

**Figure 6:**
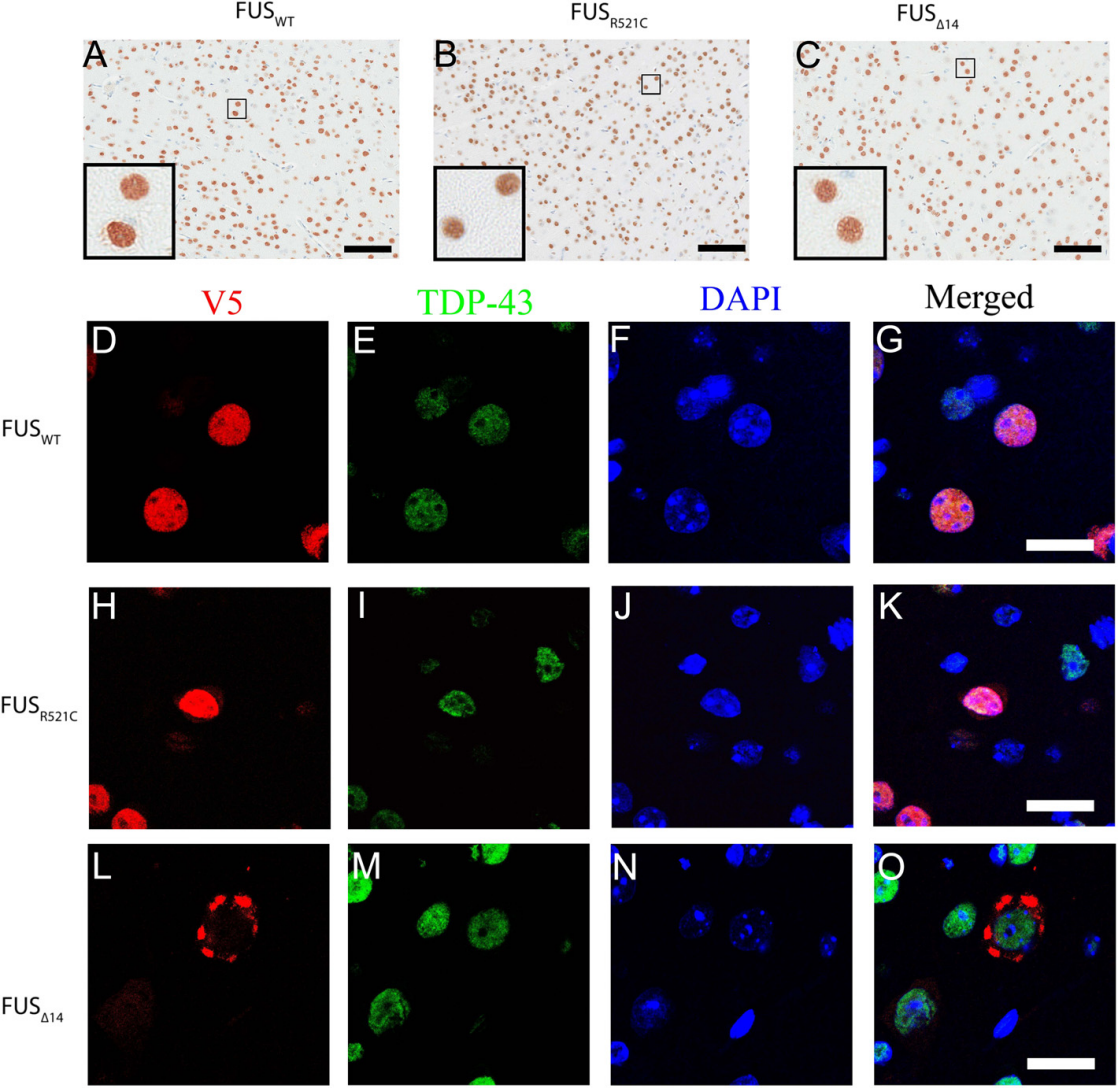
**Endogenous TDP-43 is not redistributed in FUS**_**WT,**_**FUS**_**R521C**_**and FUS**_**Δ14**_**mouse brain.** Mouse TDP-43 is predominantly in the nucleus (DAB immunohistochemistry; **A**, **B**, and **C**)**.** Double-label immunofluorescence of TDP-43 and FUS_wt_, FUS_R521C_ or FUS_Δ14_ mice (anti-V5; D-O). FUS is distributed to the nucleus in FUS_WT_ mice (**D**), increased in the neuronal cytoplasm in FUS_R521C_ mice (**H**), and accumulates as inclusions in the neuronal cytoplasm in FUS_Δ14_ mice (**L**). TDP-43 staining is nuclear in FUS_WT,_ FUS_R521C_ and FUS_Δ14_ mice (**E**, **I** and **M**). Nuclei were counterstained with DAPI. *Scale bar*: 20 μm.

Because FUS and TDP-43 have such striking structural and functional similarities, the relationship between their pathology and disease mechanism is an important unanswered question in the field. We did not find any evidence of TDP-43 redistribution from the nucleus to the cytoplasm or presence of TDP-43 within NCI in FUS_WT,_ FUS_R521C_ and FUS_Δ14_ mice (Figure 6A, B and C). In contrast, over expression of TDP-43 with a mutated nuclear localization signal in mice using SBT leads to cytoplasmic accumulation of TDP-43 (Additional file 7: Figure S7). Double-label immunofluorescence data also showed TDP-43 predominantly distributed in the nucleus in eGFP, FUS_WT,_ FUS_R521C and_ FUS_Δ14_ mice, even in neurons with well-defined NCIs (Additional file 8: Figure S8 and Figure 6C-J).

## Discussion

Our study is the first to use SBT to model FUS gene mutations in the mammalian central nervous system. The SBT paradigm was chosen because 1) mice can be generated quickly (a few months) compared to traditional transgenic techniques (a few years), 2) gene expression reaches a maximum ~3 weeks after birth, potentially avoiding toxicity during development, as has been recently observed for TDP-43 [24], and 3) recombinant AAV vectors can be rapidly generated to test different constructs *in vivo*, such as alternative promoters or putative disease associated mutations.

A key question in the field is how mutations in FUS cause neurodegeneration in ALS or FTD. Different pathogenic mechanisms for FUS mutants including toxic gain-of-function, loss-of-function, or a combination of effects have been hypothesized [10,25,26]. The SBT FUS mice we have described provide additional insight into this issue. Over expression of either FUS_WT_, FUS_R521C_, or FUS_Δ14_ was not overtly toxic to mice on an organismal level after 3 months. Similarly, transgenic rats expressing wild-type human FUS do not have acute neuronal degeneration or behavioural impairment up to the first year of life; although transgenic lines expressing FUS_R521C_ have rapid motor impairment and neuronal degeneration [27]. Despite this ALS-like phenotype, FUS R521C rat lines did not have classic neuropathology associated with FUS proteinopathies. Intriguingly, both FUS WT and R521C rats accumulated ubiquitin; however FUS did not co-localize with ubiquitin and there was no formation of distinct NCI [27]. Similar to this result we did not detect NCI in our FUS_R521C_ mice. In contrast, SBT generated FUS_Δ14_ mice have FUS and ubiquitin positive NCI, suggesting that we observed a much greater accumulation of neuropathology due to the use of this mutation, which causes a dramatic redistribution of FUS into the cytoplasm [16]. One deficiency of the SBT FUS_R521C_ or FUS_Δ14_ mice we have described is the lack of a motor phenotype or neurodegeneration. A simple explanation is that neuronal death is not present at the three-month time point we have examined. Larger cohorts of SBT FUS mice are being generated and aged to answer this question.

To date 46 mutations in FUS that are associated with ALS or FTD have been discovered, but the mechanism of their toxicity is still being deciphered [26,28,29]. A majority of these mutations cluster in or near the C-terminal PY-NLS signal, and a number of groups have now reported that in cell culture these mutations inhibit nuclear import of FUS to varying levels and increase cytoplasmic levels of FUS [27,30-32]. Our data provide the first *in vivo* evidence in mouse neurons that both ALS mutations studied, FUS_R521C_ and FUS_Δ14_, translocate to the cytoplasm at higher levels compared to control. FUS_Δ14_, which lacks the entire PY-NLS domain, had the highest levels of FUS in the neuronal cytoplasm, lowest levels in the nucleus, and was the only mutation that developed robust inclusions and insoluble FUS. The degree of FUS re-localization caused by a mutation and age of disease onset has been interpreted to mean that cytoplasmic accumulation of FUS is a primary event that drives neurodegneration [23]. Experiments in yeast [33,34], *Drosophila*[35-38], and *C. elegans*[39,40] support the concept that cytoplasmic accumulation of FUS is toxic. In contrast, Xia et al. have reported that FUS toxicity in *Drosophila* requires nuclear localization [41]. Our observation that FUS_Δ14_, which produces the earliest disease onset in humans, accumulates at the highest levels in the cytoplasm and rapidly induces multiple pathological features of FUS proteinopathies, broadly supports the hypothesis that cytoplasmic FUS is toxic . Further experiments will be necessary to dissect whether chronic cytoplasmic accumulation of FUS_Δ14_ in our mice leads to neurodegeneration and if so by what molecular mechanism.

Many neurodegenerative diseases have NCI or glial inclusions and the identity of the aggregated molecule(s) has proven to be a useful tool to characterize disease sub-types and help define disease pathogenesis [42]. Despite the lack of an obvious motor or behavioural phenotype, the SBT FUS_Δ14_ mice recapitulate many key features of FUS proteinopathies [10,19]. The most striking feature in FUS_Δ14_ mice is the robust formation of NCIs, which are immunopositive for FUS, ubiquitin, PABP1and p62/SQSTM1. NCIs containing ubiquitin and p62 are common to all sub-types of FTD and ALS-FUS. More informative is the frequent presence of basophilic NCI in FUS_Δ14_ mice, which are numerous in BIBD cases, but also present in aFTLD-U and NIFID to a lesser extent [19]. Basophilic staining of NCIs has recently been linked to the presence of RNA and RNA-binding proteins, which is logical based on the function of FUS [21]. In contrast, we only detected infrequent α-internexin staining of NCI. This may indicate that FUS_Δ14_ pathology more closely resembles BIBID and aFTLD-U. Alternatively, NCI formation may start with FUS aggregation and accumulation of α-internexin is a downstream event. Further, when FUS_Δ14_ NCIs do stain with α-internexin, it is only a portion of the total inclusion (see Figure 3 and 5). We also asked if OPTN occurred in FUS NCI based on recent reports that OPTN is a prominent marker of NCI in a subset of ALS and FTLD [43,44]. In our FUS_Δ14_ mouse model, only a small percentage of neurons had small extra-nuclear aggregates of OPTN and these did not robustly overlap with NCI detected by FUS and ubiquitin immunohistochemistry (Additional file 5: Figure S5). α-internexin inclusions were more frequent and distinct than OPTN, but still only labelled a fraction of the total FUS positive NCIs (Figure 3 and Figure 5). This observation is reminiscent of recent pathological studies of NIFID cases which found that many NCI were immunoreactive for FUS and in some cases FUS-immunoreactive NCI were more numerous than α-internexin immunoreactive NCI [17,45]. The lack of α-internexin or OPTN positive NCI in FUS_WT_ or FUS_R521C_ mice implies that inclusion formation is a requirement for the development of this pathology. Based on these findings, and the referenced pathological findings in human cases, we suggest that α-internexin and OPTN pathology are downstream events and are not a major driver of pathology and neurodegeneration in most FUS proteinopathies. Electron microscopy of NIFID tissue supports the idea that neuronal intermediate filament accumulates following FUS aggregation in the cytoplasm [46]. On-going experiments with FUS_Δ14_ mice will address whether aging increases the amount of α-internexin staining.

An interesting question raised by our data is the identity of the ubiquitinated protein(s) in FUS_Δ14_ inclusions. Ubiquitin is the most enriched marker, besides FUS, in the NCI of FUS_Δ14_, but we do not detect mono or poly-ubiquitination of FUS. This data is in agreement with multiple reports that aggregated FUS isolated from human brain is not modified by post-translational modifications, such as ubiquitin or phosphorylation [2,46-48]. Taken together, we hypothesize that accumulation of FUS_Δ14_ into NCI recruits other protein(s) that are ubiquitinated. The identity of these proteins remains to be determined and may reveal additional insights into FUS pathogenesis.

PABP-1 was another protein frequently detected in FUS_Δ14_ NCI. PABP-1 binds the poly(A) tail of mRNA and is involved in multiple steps of mRNA metabolism, including pre-mRNA splicing and regulation of translation. PABP-1 has recently gained attention in the neurodegeneration field due to its involvement in the formation of stress granules. Stress granules are dense cytoplasmic foci composed of non-translated messenger RNA, ribonucleoproteins, and other proteins that vary depending on the cell type and stress inducer [49]. Stress granules are thought to protect mRNA from harmful conditions or serve as a mechanism to rapidly modulate the types and quantities of mRNA in response to changes in the environment [50]. PABP-1 is one of the more common RNA-binding proteins that reliably associates with the various types of stress granules and is therefore commonly used as a specific marker [49]. PABP-1 labels NCI in ALS-FUS with a R521C mutation, as well as NCI in FTLD-FUS, BIBD and NIFID [31]. In cell culture, mutation of the PY-NLS can efficiently redistribute FUS into the cytoplasm, but an additional stressor appears necessary to induce localization to stress granules [31]. This finding lead the authors to speculate that two hits may be necessary to induce abnormal accumulation of FUS into stress granules and eventually end-stage NCIs [31]. This does not appear to be the case in the FUS_Δ14_ mice, because we observe numerous NCIs that co-localize with ubiquitin, p62, and PABP-1. However, milder mutations such as FUS R521C or sporadic cases may indeed require additional genetic or environmental factors to induce abnormal FUS pathology.

A major difference between the FUS positive NCI found in ALS-FUS or FTLD-FUS is that they are much larger and more insoluble than the stress granules observed in cell culture. More detailed examination of the spectrum of FUS_Δ14_ transduced neurons reveals a spectrum of aggregates ranging from multiple small foci in a neuron to a single large inclusion filling the cell body (Additional file 5: Figure S5). We hypothesize that FUS-immunoreactive inclusions evolve in stages, and may represent a transition from stress granules, which are reversible and can rapidly be dissolve, to the large, insoluble, basophilic inclusions found in end-stage FUS pathology.

Mutations in FUS were first identified in ALS cases because sequencing of the FUS gene was prioritized based on its functional similarity to TDP-43, another RNA-binding protein that had been discovered to harbour causative mutations in ALS patients. Abnormal function of FUS, TDP-43, and other RNA-binding proteins has been recently proposed to be part of a common pathway linking defects in RNA quality control to neurodegeneration in ALS and FTLD [51]. Therefore it is imperative to determine if FUS and TDP-43 share pathogenic mechanisms or interact in some way. To date, most ALS cases with FUS mutations or FTLD cases with FUS pathologies do not show abnormal TDP-43 redistribution or pathology, although one group has reported co-deposition of both proteins in NCIs [18,52]. Experiments in *Drosophila* imply that both proteins share a common pathway, with FUS acting downstream of TDP-43 [25]. Other model systems suggest that FUS and TDP-43 act through distinct pathways and cause disease through independent mechanisms, but a consensus has not yet been reached in the field [28,53,54]. We find no evidence of TDP-43 redistribution into the cytoplasm or co-aggregation into NCI in any of the FUS mice examined, even in the presence of NCIs (Figure 5). Thus in our mouse model, FUS and TDP-43 aggregation appear distinct, and lead us to speculate that despite their many similarities [6], FUS and TDP-43 have unique biological functions and their dysfunction may cause neurodegeneration through RNA dysfunction, but the precise targets and pathways are distinct.

## Conclusions

We find that SBT is a viable and rapid method to investigate the mechanism and disease relevance of genes in the nervous system of mice. The rAAV-1 vector we used in this study targets gene expression to neurons, but other rAAV vectors and promoter combinations are available to target expression to most cell types in the CNS [55]. We find that expression of a disease-associated FUS mutation (FUS_Δ14_) validates it as a pathogenic mutation, because expression of this mutation produced a number of pathological features of FUS proteinopathies. The finding that FUS_Δ14_ expression can reproduce many pathologic features observed in subtypes of FTLD and ALS FUS proteinopathies was surprising, and provides additional evidence that these diseases may share a common disease mechanism.

Overexpression of human FUS_WT_ did not induce neurodegeneration or abnormal neuropathology. Expression of the ALS mutation FUS_R521C_ was also not obviously toxic to animals at 3 months. Although FUS_R521C_ mice did not have distinct NCI, they did have a large increase in the amount of FUS present in the cell bodies and processes of neurons, as well as accumulation of biochemically insoluble FUS. The presence of aggregated FUS in FUS_R521C_, mice but no detectable NCI may indicate that we have captured an early stage of the disease process before inclusions form. Alternatively, the insoluble nature of a portion of FUS_R521C_ may indicate that small NCIs or oligomers of FUS may already be present in these animals, but are not detectable using classic immunohistochemistry. Experiments are on-going to examine behavioural and neuropathic changes overtime in SBT FUS mice.

In summary, our data supports the hypothesis that many ALS/FTD-linked mutations cause disease by increasing the cyotplasmic levels of FUS, with unknown consequences. One possibility is that cytoplasmic FUS recruits other RNA-binding proteins, such as TAF15 and PABP-1, into stress-granule like aggregates that overtime coalesce into permanent, insoluble inclusions (Additional file 5: Figure S5). Sequestration of RNA-binding proteins could dramatically affect RNA metabolism and would have devastating effects on numerous cellular events. The recent identification of an expanded hexanucleotide repeat in C9ORF72 as a frequent cause of the ALS/FTD clinical spectrum in addition to causative mutations in RNA-binding proteins, including TDP-43, FUS, sentaxin, and angiogenin, strongly implicates defects in RNA metabolism as a critical pathogenic pathway in both ALS and FTD [29,56-59]. The SBT FUS mice described in this manuscript will provide a valuable platform for further dissecting the pathogenic mechanism of FUS mutations, define the relationship between FTD and ALS-FUS, and help identify therapeutic targets that are desperately needed for these devastating neurodegenerative disorders.

## Methods

### Cloning

The generation of the N-terminally V5 tagged FUS constructs, AAV1-wild type human FUS (FUS_WT_), AAV1-human pR521C mutant FUS (FUS_R521C_) and AAV1-human p.G466VfsX14 truncated FUS (FUS_Δ14_) was previously described [16]. Inserts were cloned into the AAV1-vector using *BamH*I and *Xho*I restriction sites. The V5 tag does not alter the normal location or function of FUS and was added to the N-terminus of all constructs to facilitate detection and analysis without interference from endogenous FUS protein [32]. The sequences of all AAV1-FUS expression constructs were confirmed by direct sequencing of the complete cDNA inserts and flanking vector sequences.

### AAV1 generation and injection

All experiments with mice were approved by the Emory University and Mayo Clinic Institutional Animal Care and Use Committees and performed according to the guidelines for the care and use of laboratory animals. Animals were housed under circadian conditions and had free access to food and water. Recombinant adeno-associated virus serotype 1 (AAV1) generation and neonatal injection procedures were previously described [60]. Briefly, through viral transduction of the neuron, the protein of interest is expressed under the control of the cytomegalovirus enhancer/chicken β-actin promoter. P0 mouse pups (0–12 hours old) were cryoanesthetized on ice and bilaterally injected with 2 μl of virus (10^13^ particles/ml) per cerebral ventricle. After injection, the pups were wrapped in cage bedding, recovered on a heating pad then returned to their mother. Three groups of wild type B6C3F1 mice were injected with virus encoding FUS_WT_ (n=9), FUS_R521C_ (n=16) and FUS_Δ14_ (n=11).

### Tissue preparation

Brains were harvested at 3 months of age, except the hTDP43_M337V_ mice, which were sacrificed at 3 weeks of age. Half of the brain was immersion fixed in 4% paraformaldehyde for 24 hours and washed in Tris Buffered Saline (TBS). Sections were embedded in paraffin, sectioned in the sagittal plain (5 μm thick) and mounted on glass slides. The other half of the brain was frozen on dry ice for biochemistry.

### Histology and immunohistochemistry

Sections were deparaffinized in xylene and rehydrated in a graded series of alcohol followed by dH_2_O. Antigen retrieval was performed in a dH_2_O steam bath for 30 minutes. Immunohistochemistry was performed on an automated stainer (DAKO Auto Machine Corporation) and the DAKO EnVision+ HRP system. All sections were briefly counterstained with hematoxylin. For Double labelling immunofluorescence.

After deparaffinised and rehydrated, sections were incubated in retrieval solution (DAKO) for 30 min at 95 degree. Tissues were immunostained with the following primary antibodies at the indicated concentrations: V5 (monoclonal, Invitrogen, 1:1,000 and polyclonal, Bethyl Labs, 1:1000), FUS (polyclonal, Sigma, 1:2,500), TDP-43 (monoclonal human specific, Novus Biologicals, 1:3,000 and polyclonal, Proteintech, 1:5,000), PABP1 (polyclonal, Cell signaling, 1:100), α-internexin (monoclonal IgG; from Gerry Shaw, University of Florida, 1:50), OPTN (polyclonal, Abcam, 1:100) and ubiquitin (monoclonal, Millipore,1:60K and polyconal, DAKO, 1:200), TAF15(polyclonal, Bethyl Lab, 1:250), P62(monoclonal, BD Biosciences, 1:100), NeuN(polyclonal, Millipore, 1:200), IBA1(polyclonal, WAKO, 1:1000), EWS(polyclonal, Epitomics, 1:250), GFAP(polyclonal, Millipore, 1:1000). For Double labelling immunofluorescence, the sections were incubated in the secondary antibodies conjugated to Cy3 and fluorescein(1:250, Invitrogen). Hematoxylin-eosin (H&E) staining was performed on paraffin sections. Stained sections were captured using the ScanScope XT image scanner (Aperio, Vista, CA, USA) and processed with ImageScope software. Ubiquitin accumulation was quantified using the ImageScope positive pixel count algorithm for DAB staining (Aperio). Other photomicrographs were captured on an Olympus BX50 microscope with DP12 digital camera (Olympus, PA, USA). Confocal images were collected with a Zeiss LSM 510 NLO META system.

### Biochemistry

Nuclear and cytoplasmic enriched protein fractions were isolated from the tissue using the ProteoJET Cytoplasmic and Nuclear Extraction Kit (Fermentas, Ontario, Canada) following the manufacturer’s protocol. Protein separation and immunoblot were performed as previously described [61,62]. Briefly, proteins were separated on 4-12% Bis-Tris XT gels (Bio-Rad, CA, USA) with XT-MES running buffer and transferred to a 0.2μm nitrocellulose membrane. After overnight blocking at 4°C in a 0.5% casein block solution, blots were probed with anti-V5 (monoclonal, Invitrogen, 1:1K) and anti-histone 3 (polyclonal, Cell Signaling, 1:1,000) primary antibody, followed by horseradish peroxidase (HRP)-conjugated secondary antibody against mouse and rabbit IgG (Jackson Immuno Research, 1:5K). Relative band intensity was quantified using ImageJ software (NIH).

## Abbreviations

ALS: Amyotrophic lateral sclerosis; FTD: Fronto-temproal dementia; FTLD: Frontotemporal lobar degeneration; aFTLD-U: Atypical FTLD with ubiquitinated inclusions; NIFID: Neuronal intermediate filament inclusion disease; BIBD: Basophilic inclusion body disease; FUS or TLS: Fused in sarcoma or translocated in sarcoma; NCIs: Neuronal cytoplasmic inclusions; NLS: Nuclear localization sequence; NES: Nuclear export signal; SBT: Somatic brain transgenesis; AAV: Adeno-associated virus; PABP-1: Polyadenylate-binding protein 1.

## Competing interests

The authors declare that they have no conflict of interest.

## Authors’ contributions

CV, QD, PD, and TK carried out animal experiments, western blots, and analysis of data. MDH, JK, and CCD cloned constructs and produced AAV. CV, QD, GT, DWD, and TK performed tissue immunostaining and analysis. TG, PD, RR, DWD, and TK conceived of the study, participated in design, experiment coordination, and data analysis. CV, QD, and TK drafted the manuscript. All authors read and approved the final manuscript.

## Supplementary Material

Additional file 1 **Figure S1.** Neuron-specific expression of FUS in mouse cortex and hippocampus. Confocal imaging shows that cells transduced with rAAV1 expressing FUS_WT_ (anti-V5; A, E, I, and M_)_ colocalize with the neuronal marker NeuN (B and F) but not an astrocyte marker (GFAP; J and N) in cortex and hippocampus. Nuclei were counterstained with DAPI (C, G, K and O). Scale bar: 50μm. Click here for file

Additional file 2 **Figure S2.** Mutation-dependent redistribution of FUS. Double-immunofluorescence staining of V5 and a neuronal marker NeuN in FUS_WT_, FUS_R521C_, and FUS_Δ14_ mice (A-L). V5 staining mainly located in nucleus in FUS_WT_ mouse (A-D). Strong nuclear and some cytoplasmic V5 staining in FUS_R521C_ mouse neurons (E-H). Cytoplasmic accumulation of V5 staining in FUS_Δ14_ mice (I-L). Scale bar:10μm. Click here for file

Additional file 3 **Figure S3.** No marked astrocytosis or microglial activation in FUS_Δ14_ mice. Double-label immunofluorescence of a microglia marker IBA-1and V5 in the cerebral cortex of FUS_WT_ and FUS_Δ14_ mice (A-H). Double-label immunofluorescence of a astrocyte marker GFAP and V5 in cerebral cortex of FUS_WT_ and FUS_Δ14_ mice (A-H). Nuclei were counterstained with DAPI (C, G, K, and O). *Scale bar*: 50 μm. Click here for file

Additional file 4 **Figure S4.** Quantification of ubiquitin levels in SBT FUS mice. The levels of ubiqutin immunoreactivity in the brain of FUS_WT_, FUS_R521C_, and FUS_Δ14_ mice were quantified using positive pixel counts (default strong positive DAB threshold) and analyzed relative to the total pixels in the analysis area using the ImageScope software (Aperio). Compared to FUS_WT_ and FUS_R521C_ mice, FUS_Δ14_ mice have significantly greater accumulation of ubiquitin (***p < 0.001, One-way analysis of variance; Graph Pad Prism 5). Values represent mean ± SEM (n=6 for each experimental group). Click here for file

Additional file 5 **Figure S5.** Diversity of neuronal cytoplasmic inclusions in the brains of FUS_Δ14_ mice. The different sizes of inclusions may represent a spectrum of growth from small aggregates to large, insoluble NCIs. The first phase is characterized by nuclear and cytoplasmic location of FUS (A), followed by formation of small, round shaped aggregates (B). FUS accumulates in the cytoplasm (darker staining), associated with depletion of FUS from the nucleus (lighter staining), and the aggregates eventually merge into one or two amorphous inclusions (C). In the end stage, the NCI’s are round or oval shaped and FUS is no longer localized in the nucleus. (D). Click here for file

Additional file 6 **Figure S6.** Optineurin is not a robust marker of NCI in FUSs_Δ14_ mice. Immunohistochemistry of cerebral cortex of FUS_WT_ (**A**), FUS_R521C_ (**B**) and FUS_Δ14_ (**C**) mice shows no optineurin (OPTN) positive inclusions. There are occasional neurons in the cortex of FUS_Δ14_ mice with small extranuclear protein aggregates that are positive for OPTN (*arrows***C**). Detection of endogenous mouse optineurin with this antibody was weak, making it difficult to draw definitive conclusions about co-localization with FUS-positive NCI. Scale bar: 50 μm. Click here for file

Additional file 7 **Figure S7.** Expression of human TDP-43 with a NLS mutation in mouse brain using SBT leads to increased cytoplasmic levels in mouse neurons. Mutant human TDP-43_NLS_ accumulates in the soma, dendrites, and axons of neurons. *Scale bar*, 100 μm. Click here for file

Additional file 8 **Figure S8.** Expression of eGFP in mouse brain using SBT did not lead to redistribution of TDP-43. Double labeling of EGFP (A) and TDP-43(B). Scale bar: 20μm. Click here for file
