# Supplementary figures and images for: Expression of Fused in sarcoma mutations in mice recapitulates the neuropathology of FUS proteinopathies and provides insight into disease pathogenesis
Source: Mol Neurodegener. 2012 Oct 10;7:53. doi: 10.1186/1750-1326-7-53 (PMC3519790; doi:10.1186/1750-1326-7-53)

V5

NeuN

DAPI

Merged

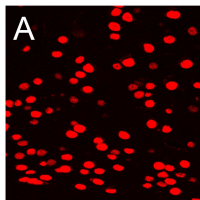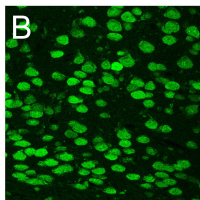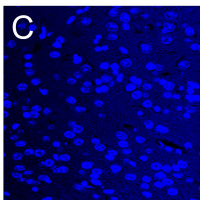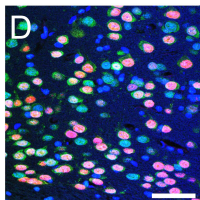

Cortex

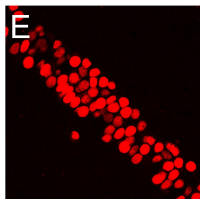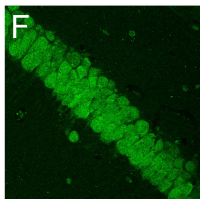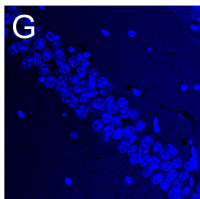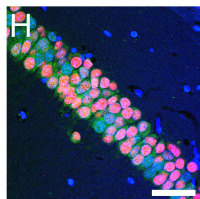

Hipp

V5

GFAP

DAPI

Merged

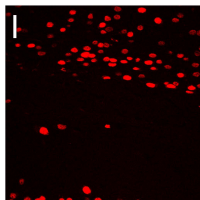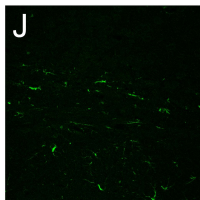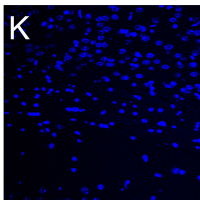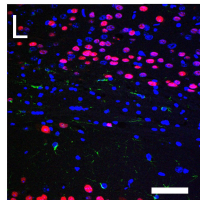

Cortex

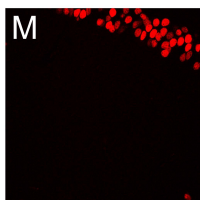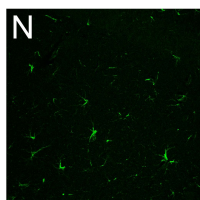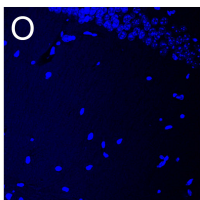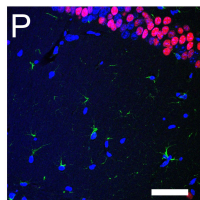

Hipp

Supplement: Additional file 1 — Figure S1. Neuron-specific expression of FUS in mouse cortex and hippocampus. Confocal imaging shows that cells transduced with rAAV1 expressing FUSWT (anti-V5; A, E, I, and M) colocalize with the neuronal marker NeuN (B and F) but not an astrocyte marker (GFAP; J and N) in cortex and hippocampus. Nuclei were counterstained with DAPI (C, G, K and O). Scale bar: 50μm. [file 1750-1326-7-53-S1.pdf]

V5

NeuN

DAPI

Merged

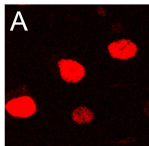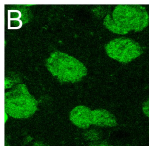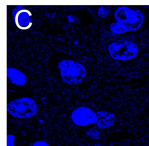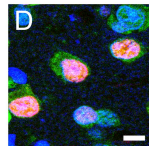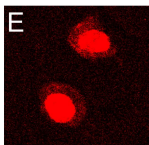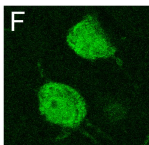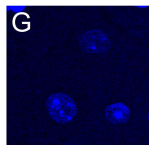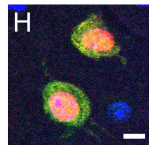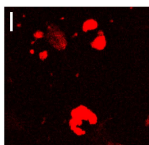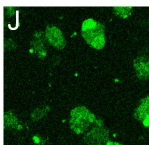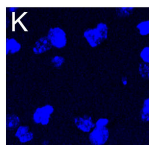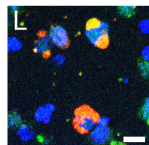FUS<sub>WT</sub>FUS<sub>R521C</sub>FUS<sub>Δ14</sub>

Supplement: Additional file 2 — Figure S2. Mutation-dependent redistribution of FUS. Double-immunofluorescence staining of V5 and a neuronal marker NeuN in FUSWT, FUSR521C, and FUSΔ14 mice (A-L). V5 staining mainly located in nucleus in FUSWT mouse (A-D). Strong nuclear and some cytoplasmic V5 staining in FUSR521C mouse neurons (E-H). Cytoplasmic accumulation of V5 staining in FUSΔ14 mice (I-L). Scale bar:10μm. [file 1750-1326-7-53-S2.pdf]

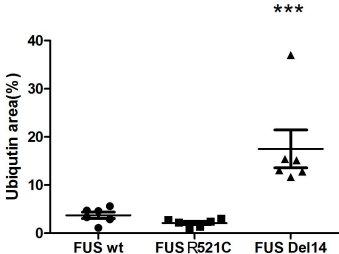

Supplement: Additional file 4 — Figure S4. Quantification of ubiquitin levels in SBT FUS mice. The levels of ubiqutin immunoreactivity in the brain of FUSWT, FUSR521C, and FUSΔ14 mice were quantified using positive pixel counts (default strong positive DAB threshold) and analyzed relative to the total pixels in the analysis area using the ImageScope software (Aperio). Compared to FUSWT and FUSR521C mice, FUSΔ14 mice have significantly greater accumulation of ubiquitin (***p < 0.001, One-way analysis of variance; Graph Pad Prism 5). Values represent mean ± SEM (n=6 for each experimental group). [file 1750-1326-7-53-S4.pdf]

FUS<sub>Δ14</sub>

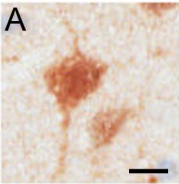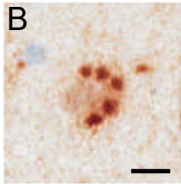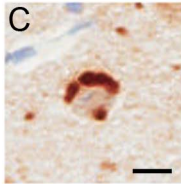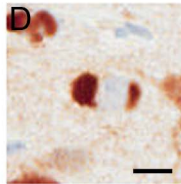

Supplement: Additional file 5 — Figure S5. Diversity of neuronal cytoplasmic inclusions in the brains of FUSΔ14 mice. The different sizes of inclusions may represent a spectrum of growth from small aggregates to large, insoluble NCIs. The first phase is characterized by nuclear and cytoplasmic location of FUS (A), followed by formation of small, round shaped aggregates (B). FUS accumulates in the cytoplasm (darker staining), associated with depletion of FUS from the nucleus (lighter staining), and the aggregates eventually merge into one or two amorphous inclusions (C). In the end stage, the NCI’s are round or oval shaped and FUS is no longer localized in the nucleus. (D). [file 1750-1326-7-53-S5.pdf]

FUS<sub>WT</sub>

FUS<sub>R521C</sub>

FUS<sub>Δ14</sub>

OPTN

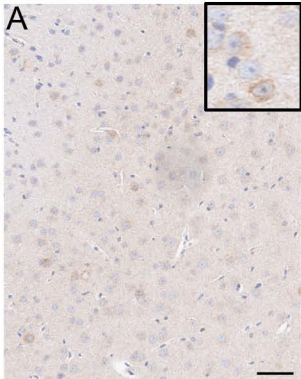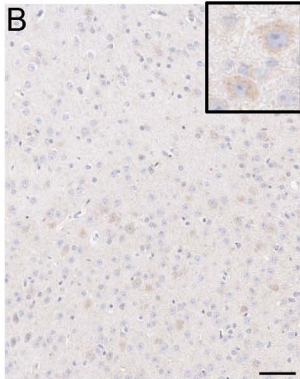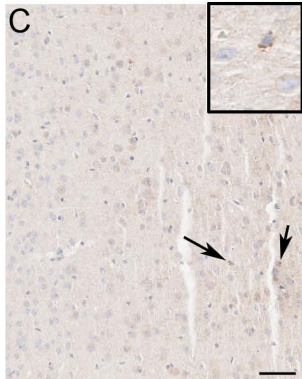

Supplement: Additional file 6 — Figure S6. Optineurin is not a robust marker of NCI in FUSsΔ14 mice. Immunohistochemistry of cerebral cortex of FUSWT (A), FUSR521C (B) and FUSΔ14 (C) mice shows no optineurin (OPTN) positive inclusions. There are occasional neurons in the cortex of FUSΔ14 mice with small extranuclear protein aggregates that are positive for OPTN (arrowsC). Detection of endogenous mouse optineurin with this antibody was weak, making it difficult to draw definitive conclusions about co-localization with FUS-positive NCI. Scale bar: 50 μm. [file 1750-1326-7-53-S6.pdf]

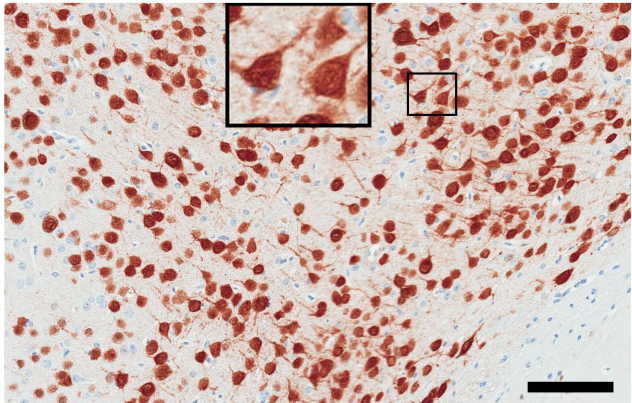

Supplement: Additional file 7 — Figure S7. Expression of human TDP-43 with a NLS mutation in mouse brain using SBT leads to increased cytoplasmic levels in mouse neurons. Mutant human TDP-43NLS accumulates in the soma, dendrites, and axons of neurons. Scale bar, 100 μm. [file 1750-1326-7-53-S7.pdf]

EGFP

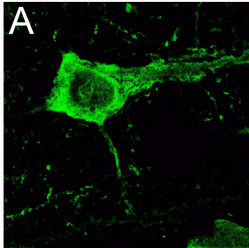

TDP-43

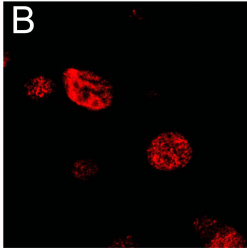

DAPI

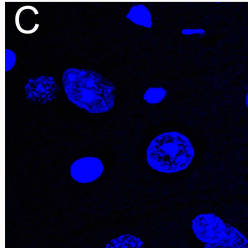

Merged

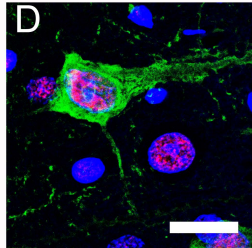

Supplement: Additional file 8 — Figure S8. Expression of eGFP in mouse brain using SBT did not lead to redistribution of TDP-43. Double labeling of EGFP (A) and TDP-43(B). Scale bar: 20μm. [file 1750-1326-7-53-S8.pdf]
