# Supplementary material for: Expression of Fused in sarcoma mutations in mice recapitulates the neuropathology of FUS proteinopathies and provides insight into disease pathogenesis
Source: Mol Neurodegener. 2012 Oct 10;7:53. doi: 10.1186/1750-1326-7-53 (PMC3519790; doi:10.1186/1750-1326-7-53)
Supplement: Additional file 3 — Figure S3. No marked astrocytosis or microglial activation in FUSΔ14 mice. Double-label immunofluorescence of a microglia marker IBA-1and V5 in the cerebral cortex of FUSWT and FUSΔ14 mice (A-H). Double-label immunofluorescence of a astrocyte marker GFAP and V5 in cerebral cortex of FUSWT and FUSΔ14 mice (A-H). Nuclei were counterstained with DAPI (C, G, K, and O). Scale bar: 50 μm. [file 1750-1326-7-53-S3.pdf]

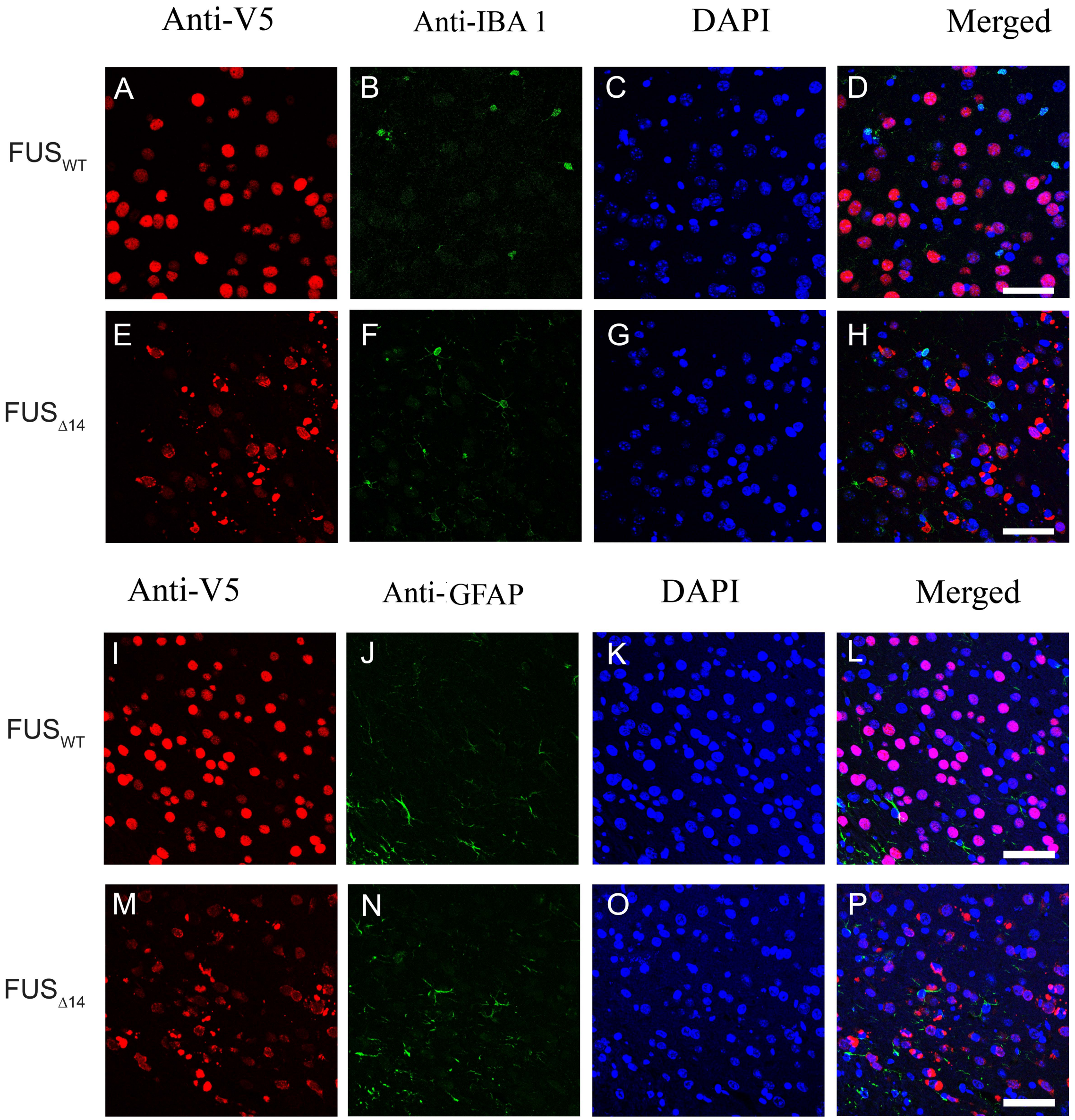

Anti-V5
Anti-IBA 1
DAPI
Merged
FUSWT
A
B
C
D
FUSΔ14
E
F
G
H
Anti-V5
Anti-GFAP
DAPI
Merged
FUSWT
I
J
K
L
FUSΔ14
M
N
O
P
